# Vascular 3D Printing with a Novel Biological Tissue Mimicking Resin for Patient-Specific Procedure Simulations in Interventional Radiology: a Feasibility Study

**DOI:** 10.1007/s10278-021-00553-z

**Published:** 2022-01-07

**Authors:** R. Kaufmann, C. J. Zech, M. Takes, P. Brantner, F. Thieringer, M. Deutschmann, K. Hergan, B. Scharinger, S. Hecht, R. Rezar, B. Wernly, M. Meissnitzer

**Affiliations:** 1 Department of Radiology, University Hospital Salzburg, Paracelsus Medical University, 5020 Salzburg, Austria; 2 Clinic of Radiology & Nuclear Medicine, University Hospital Basel, University of Basel, 4031 Basel, Switzerland; 3 Clinic for Oral and Maxillofacial Surgery, University Hospital Basel, University of Basel, 4031 Basel, Switzerland; 4 Clinic of Internal Medicine II, Department of Cardiology and Internal Intensive Care Medicine, University Hospital Salzburg, Paracelsus Medical University, 5020 Salzburg, Austria

**Keywords:** Printing, Three-dimensional, Stereolithography, Radiology, Interventional, Angiography

## Abstract

Three-dimensional (3D) printing of vascular structures is of special interest for procedure simulations in Interventional Radiology, but remains due to the complexity of the vascular system and the lack of biological tissue mimicking 3D printing materials a technical challenge. In this study, the technical feasibility, accuracy, and usability of a recently introduced silicone-like resin were evaluated for endovascular procedure simulations and technically compared to a commonly used standard clear resin. Fifty-four vascular models based on twenty-seven consecutive embolization cases were fabricated from preinterventional CT scans and each model was checked for printing success and accuracy by CT-scanning and digital comparison to its original CT data. Median deltas (Δ) of luminal diameters were 0.35 mm for clear and 0.32 mm for flexible resin (216 measurements in total) with no significant differences (*p* > 0.05). Printing success was 85.2% for standard clear and 81.5% for the novel flexible resin. In conclusion, vascular 3D printing with silicone-like flexible resin was technically feasible and highly accurate. This is the first and largest consecutive case series of 3D-printed embolizations with a novel biological tissue mimicking material and is a promising next step in patient-specific procedure simulations in Interventional Radiology.

**Supplementary Information:**

The online version contains supplementary material available at 10.1007/s10278-021-00553-z.

## Introduction

Three-dimensional (3D) printing has become an innovative way of visualizing imaging data based on computed tomography (CT) or magnetic resonance imaging (MRI) [1, 2]. Applications include surgery planning, (pre)procedural simulation, device testing, education, and more [3–6]. Several studies have shown the potential benefits and fields of application for Interventional Radiology (IR), especially for manufacturing vascular structures and the simulation of endovascular procedures [7–9]. However, the technology still has many limitations, particularly time-consuming and complex manufacturing processes and the lack of ideal vasculature-like 3D printing materials [10]. Additionally, there is a wide range of available printing technologies, post-processing, and segmentation software and simulation models [11, 12]. In short, the ideal setup for patient-specific simulations with 3D printing in IR has not yet been found.

During the past decades, multiple 3D printing technologies were developed and used in different fields of medicine. In 1984 the first patents for computer automated manufacturing processes were filed, including Hull and Arcadia with his “Apparatus for production of three-dimensional objects by stereolithography” [13]. Stereolithography (SLA) is until today the most commonly used 3D printing technology and is utilized in medicine already since the 90 s [14, 15]. It is a liquid-based additive manufacturing process using photopolymerization of resin, where an ultraviolet laser cures each layer slice-by-slice. The process starts from the bottom of the model to build the layers upward and uses support structures to stabilize the model while printing. These supports are a challenge for vascular 3D printing, because they can be difficult to remove afterward, especially when they are inside the vascular lumen (so called internal supports). Multiple other technologies were introduced including fused deposition modeling (FDM), multijet and polyjet printing (MJP, PJP), selective laser sintering (SLS), digital light processing (DLP), and colorjet printing (CJP) [4–6, 11]. However, SLA is considered to be one of the most accurate methods with the best surface finish [11].

Another challenge, besides the support structures, still remains the selection of an appropriate 3D printing material. There is a huge variety of materials with different technical properties, including also different kinds of resins for SLA printing [10, 16]. O’Reilly et al. recently published a comprehensive library of 3D printing materials that may be suitable as physical and radiological biologic mimics, but a suitable material for direct vascular 3D printing for interventional procedure simulations was not yet available [17]. So far, this issue could only be overcome with a complex and time-consuming workaround by silicone molding [10], but a recently introduced silicone-like resin with biological tissue mimicking characteristics might solve these problems.

This study evaluates the technical feasibility and accuracy of direct vascular SLA 3D printing with this new material for endovascular procedure simulations and technically compares it to a commonly used standard clear resin, based on a series of 27 consecutive real-life embolizations from our institution.

## Materials and Methods

### Patient and Imaging Data

All consecutive patients from September 2018 to March 2019 treated with selective embolization and with prior procedure-related CT examination at our institution were included in this retrospective study. A positive ethics vote was obtained (IRB-No. 1004/2020). Patient-identifiers were irreversibly anonymized and replaced by a random ID. The only inclusion criterion concerning the preprocedural CT was an axial reconstruction with 1-mm slice thickness and arterial phase contrast enhancement. All CTs were performed for procedural planning as well as for diagnostic purposes in clinical routine using 128-slice multidetector CT (Somatom Definition AS +, Siemens). A standard protocol with a bolus of 120 ml non-ionic contrast media was used with an injection rate of 3.5–5 ml/s. The arterial phase (CTA) was acquired at a delay of 30 s post-injection and axial thin-sliced reconstructions with 1-mm slice thickness were generated in soft-tissue window. All other phases like native or portal venous phase were not used and, respectively, not relevant for creating the vascular models. To investigate our manufacturing process in a clinical setting, patients were not excluded because of poor image quality (i.e., caused by artifacts, low contrast-enhancement).

### Image Processing and Segmentation

The digital models were generated based on the CT scans by segmenting the relevant arteries including the aorta, using the open-source biomedical image processing software ImageJ (Laboratory for Optical and Computational Instrumentation, University of Wisconsin). Segmentation was done case-by-case by using the window-level tool for segmenting the case-related arteries. Calcifications were subtracted with the threshold tool; consecutively only the inner vascular lumen was segmented. The surface models were generated and exported as binary stereolithography (STL) files using the integrated 3D-Viewer with standard preset settings (display as = surface, color = white, threshold = 50, resampling factor = 2). The open-source 3D graphics software Blender (Blender Foundation) was used to remove non-vascular structures from the STL-file, to smooth the model with subdivision surface modifier (algorithm = catmull–clark, iterations = 1) and to generate the vessel walls with 1-mm wall thickness, using the solidify modifier (mode = complex, thickness mode = fixed, boundary shape = none).

### 3D Printing

An in-house 3d-print lab with a SLA 3D printer (Form 3, Formlabs) at our radiology department was used to create the patient-specific vascular models. Layer thickness was set to 0.1 mm for both materials. Support structures were automatically generated and manually refined using a density of 0.8, touching point size of 0.4 mm and deactivation of internal supports. Each embolization case was printed twice, once with standard clear (transparent but rigid) and once with novel flexible resin (transparent and flexible). Post-processing included automatic model cleaning for 10 min in isopropanol (Form Wash, Formlabs), manual flushing of small vessels with isopropanol-injection by hand, removal of the support structures, and lastly, curing the model in ultraviolet light for 10 min with 40 °C (Form Cure, Formlabs). The whole manufacturing process is summarized in Table 1.

**Table 1:** The manufacturing process. Summary of the steps of our manufacturing process for vascular 3D printing with clear and flexible resin, used in this case series

Step	Description
1. CT scan	Standard protocol **CT angiography** in arterial phase with axial reconstructions in 1-mm slice thickness *Note that a larger slice thickness will cause a loss of detail and the formation of steps in the vascular models*
2. Segmentation	**ImageJ** (open-source software) was used for segmentation and the included 3D-Viewer plugin for creating surface models in form of STL files *Note that any segmentation and 3D-modelling software can be used, but has to be focused on a high grade of detail of the procedure-relevant vascular system*
3. 3D modeling	**Blender** (open-source software) was used for digital post-processing (removing residual non-vascular structures) and creating the vessel walls (1 mm) for each STL file
4. 3D printing	**PreForm** (included software, Formlabs) was used for 3D printing at a resolution of 0.1 mm and with automatic generation of external supports at a density of 0.8 and touching point size of 0.4 mm *Note that it is important that internal (intraluminal) support structures are deactivated, because they cannot be removed from the inside of small vessels afterward*
5. Cleaning	The printed models were at first **cleaned** automatically for 10 min in isopropanol (Form Wash, Formlabs) and second by hand using isopropanol in a syringe to flush and fully clean the small structures *Note that it is important that all vessels are free from residual resin to prevent permanent vessel blockages from final curing in ultraviolet light (step 7)*
6. Support removal	**Support structures** were carefully, but easily removed by hand, right after cleaning *Note that support structures are much easier and safer to remove before curing (step 7)*
7. Curing	The models were finally **cured** in ultraviolet light for 10 min with 40 °C (Form Cure, Formlabs) *Note that the models are ready to be used for simulation immediately after curing*

### Measurements

**Fig. 1:**
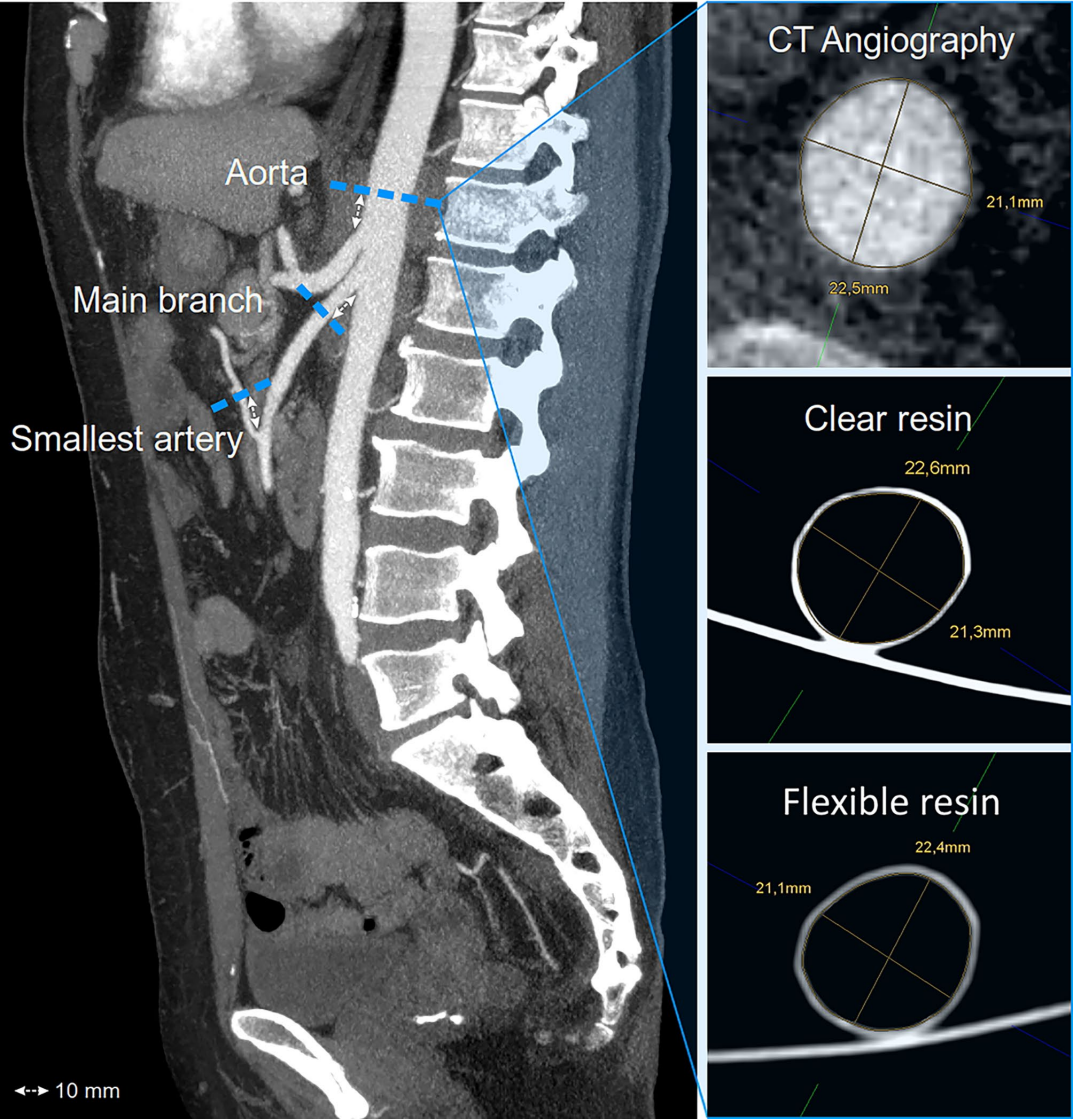
Principle of the diameter measurements of the CT-scanned vascular models and their original CT data. Each case was measured for mean luminal diameters on the patient’s CT scan as well as the related CT-scanned printed models (clear and flexible resin) at three predefined points: (1) the aorta, (2) the procedure-relevant main branch, and (3) the smallest procedure-relevant artery

All vascular models were scanned with CT and reconstructed in axial slices of 1-mm thickness (Somatom Definition AS +, Siemens). The diameters were measured orthogonal to the vessel axis using multiplanar reconstruction (MPR) in our PACS system, because the centerline method was not applicable to the printed models without contrast enhancement. All models and their related CTAs were measured at the same 3 points for their mean luminal diameters: (1) at the aorta, (2) a main branch of the aorta (i.e., celiac artery), and (3) the smallest, procedure-relevant artery. To assure that the measurements were performed at the same position, the aorta was always measured 10 mm proximal to a main branch and the main branch as well as the smallest procedure-relevant artery were always measured 10 mm distal their origin, demonstrated in Fig. 1. In case the main branch and the smallest artery were the same, for example, in a bronchial artery bleeding, the smallest procedure-relevant artery was measured twice 10 mm after origin and 10 mm before its ending. The differences between the CT-scanned models and their original CTA were calculated with divergence and absolute delta (Δ) in mm. The printing accuracy was rated based on absolute delta (Δ): a delta > 1.5 mm was considered as insufficient, 1.0 to 1.5 mm as sufficient, 0.5–1.0 mm as good, and < 0.5 mm as excellent (Table 2).

**Table 2:** Printing accuracy. The printing accuracy was rated based on the absolute delta (Δ) of the vascular model diameters compared to their originating CT scan

**Absolute delta (Δ) compared to CT scan**	**Rating of printing accuracy**
> 1.5 mm	Insufficient
1.0–1.5 mm	Sufficient
0.5–1 mm	Good
< 0.5 mm	Excellent

### Statistics

The data was assessed for normal distribution using Kolmogorov–Smirnov testing. Given the non-normal distribution, continuous variables were given in median and interquartile range (IQR). Differences across more than 2 groups were assessed using Friedman’s test and Dunn’s multiple comparison test to compare flexible and clear resin versus CT (control group). Differences between two groups were assessed using Wilcoxon matched-pair signed rank test. All tests were two-sided and *p*-values < 0.05 were considered to be statistically significant. GraphPad Prism was used to analyze and depict data.

## Results

### Patient and Imaging Data

A series of 27 consecutive embolization procedures performed at our institution over a 6-month period (18 male, 9 female, age = 65 years [18–93 years]) was available with preinterventional planning CTAs with thin-sliced axial reconstructions of 1 mm. The case series involved acute bleedings (14), aneurysms and pseudoaneurysms (5), tumor embolizations (7), and a pulmonary vascular malformation (1). All other procedures performed during this half year period did not have preinterventional CTAs for generating high-resolution 3D models and were not included in this study. TACE and SIRT, for example, usually either received preinterventional MRI or a CT from external institutions without CTA in 1-mm slice thickness and therefore did not match our inclusion criteria. All cases finally included in this study are described and listed per organ system in Table 3.

**Table 3:** The embolization case series. Overview and description of the consecutive embolization case series including 27 patients which were used to fabricate 54 vascular models with two different printing materials

**Organ/system**	**Indication**	**Cases**
Liver	Hemorrhage from tumor (1 ×), hemangioma (1 ×), and operation (2 ×)	4
Pancreas	Pseudoaneurysm in the arterial arcades of the pancreas after pancreatitis (1 ×)	1
Spleen	Splenic artery aneurysm (2 ×) and pseudoaneurysm after pancreatitis (1 ×)	3
Kidney	Hemorrhage from tumor (3 ×), renal cyst (1 ×), and operation (1 ×)	5
Gastrointestinal system	Mesenteric pseudoaneurysm (1 ×), hemorrhage from duodenal ulcer (1 ×), anticoagulation (1 ×), and GI tumor (2 ×)	5
Retroperitoneum	Hemorrhage from trauma (1 ×), anticoagulation (1 ×), and post-TAVI (1 ×)	3
Pulmonary	Hemorrhage from tumor (2 ×) and cystic fibrosis (1 ×), pulmonary arteriovenous malformation (1 ×)	3
Soft tissue	Trauma under anticoagulation (2 ×)	2
**Total**		**27**

### Manufacturing Process

**Fig. 2:**
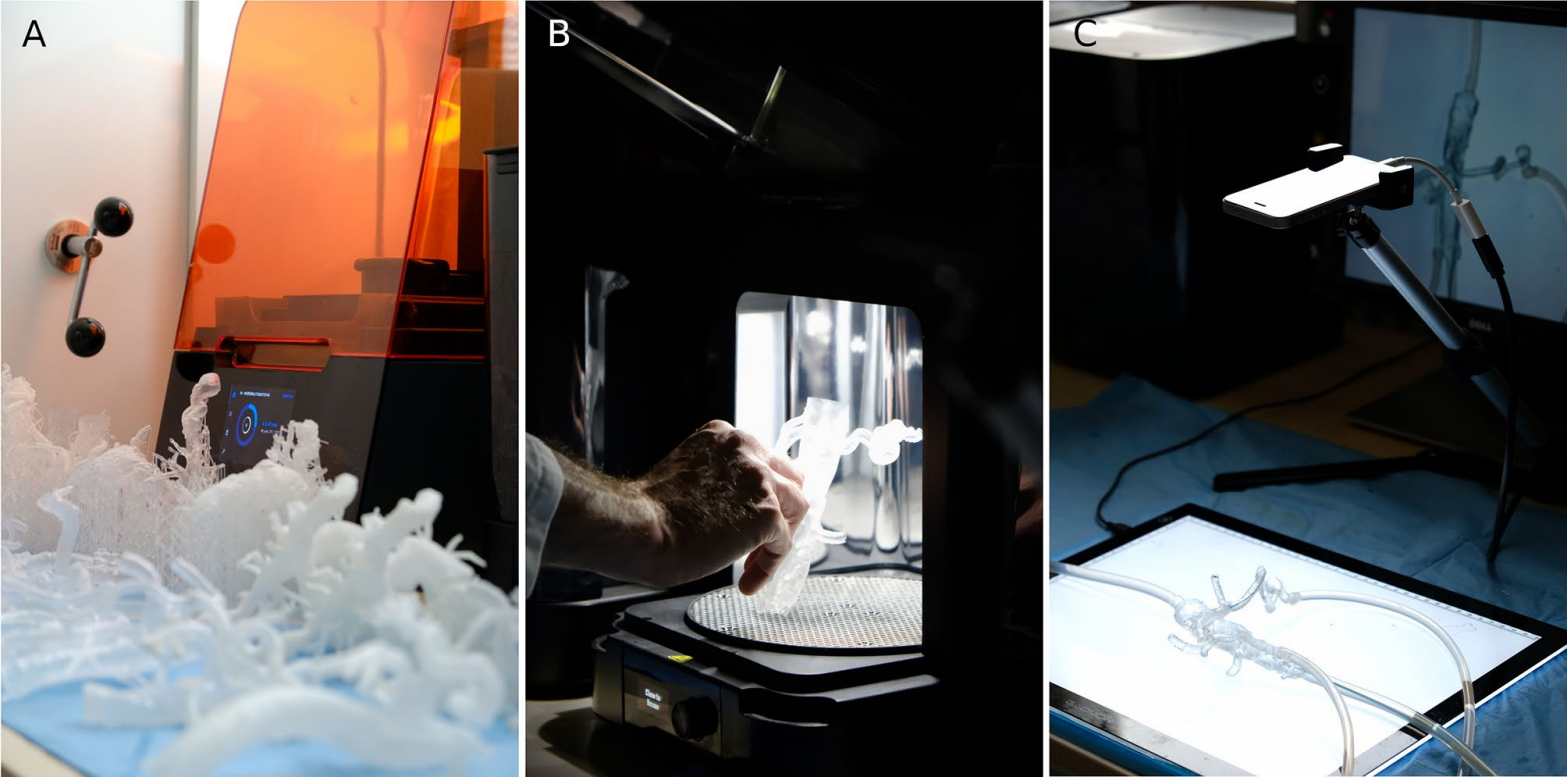
Excerpt of the manufacturing process and simulation setting. **A** 3D printer with a set of printed models in front. **B** Curing step; note that support structures are already removed. **C** Simulation setting with a smartphone camera for 2D projection and a LED panel to improve the visibility of guidewires and catheters inside the model

**Fig. 3:**
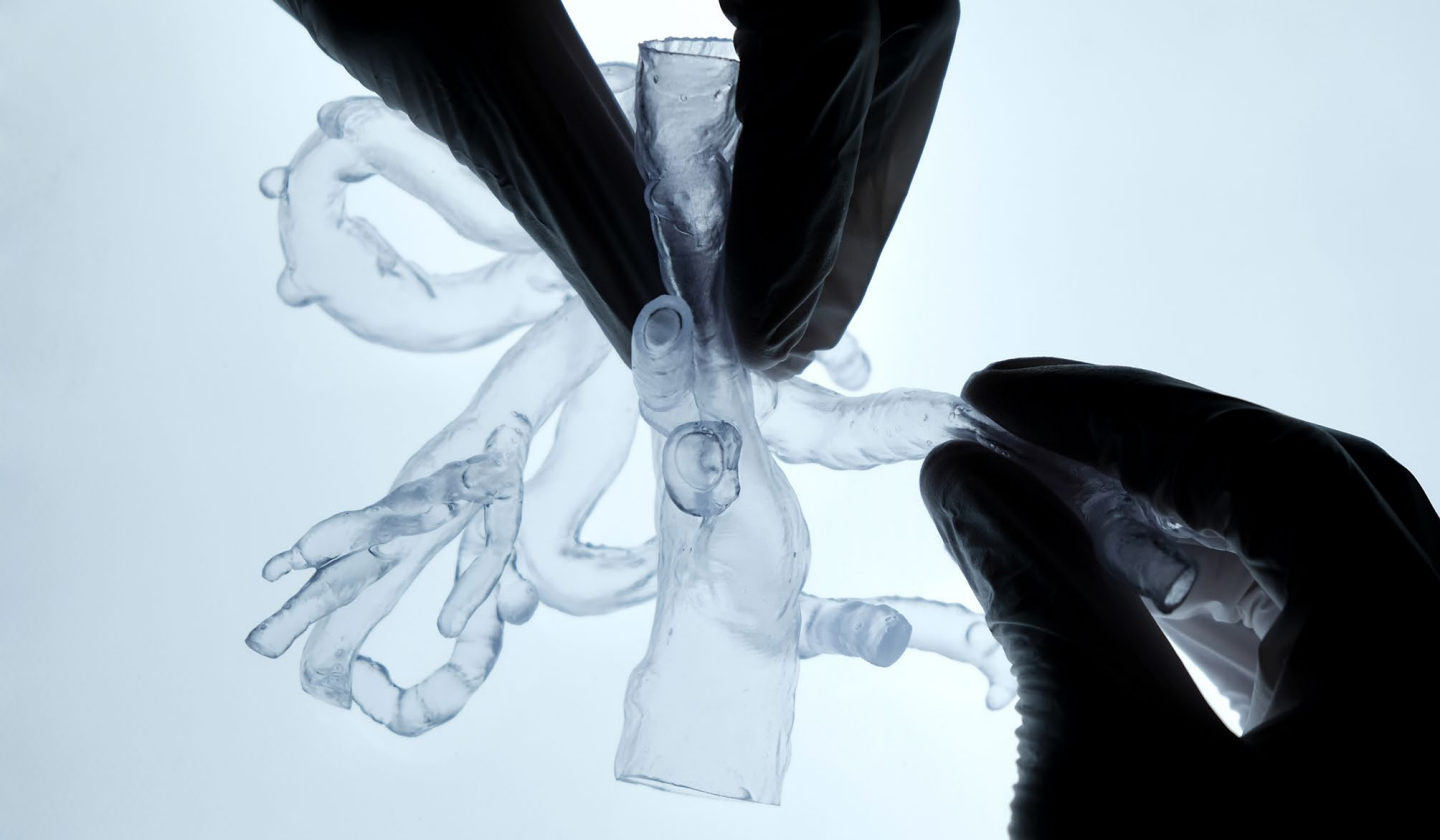
Proof of flexibility and transparency of a CT-derived vascular model, printed with flexible resin. The aorta and its branches are easily compressed with two fingers, demonstrating the flexible quality of this novel 3D-printing material. Transparency is high and can be further improved as demonstrated by background lighting with a LED panel

A total of 54 vascular models with standard clear and transparent flexible resin (50:50) was created based on the 27 IR cases. Three of 54 prints technically failed because of material rupture while printing (5.6% printing error): the pulmonary arteriovenous malformation (AVM) with flexible resin and a mesenteric artery bleeding with extensive kinking in both 3D printing materials. Segmentation failed in one case of bronchial artery bleeding, because the bronchial artery was running parallel so close to the aorta (< 1 mm) that they fused together to one vessel in both printing materials (3.7% segmentation error). Two cases had a lack of detail on the CT scan, so that the procedure-relevant artery could not be segmented for both materials (7.4% lack of detail error). In total, 45 of 54 (83.3%) vascular models were printed successfully with the procedure-relevant arteries. Separated into both printing materials and excluding the processing errors which affect both materials likewise (segmentation error and lack of detail on the CT scan), 23 of 24 cases were printed successfully with clear (96%) and 22 of 24 with flexible resin (92%). Including all technical errors, 23 of 27 prints were successful with clear (85.2%) and 22 of 27 prints with flexible resin (81.5%). The summary of the results is listed in Table 4 and the manufacturing process is demonstrated in Fig. 2. The vascular models are transparent and flexible and can be easily compressed by hand as shown in Fig. 3.

**Table 4:** Printing successResults of the manufacturing process, separated into the two printing materials clear (V4) and flexible resin (80A)

	**Clear resin (V4)**	**Flexible resin (80A)**
Print error (*n*)	1	2
Segmentation error (*n*)	1	1
Lack of detail on the CT-scan error (*n*)	2	2
Overall failed prints (*n*)	4	5
Overall successful prints (*n*)	23	22
**Overall success rate (%)**	**85.2%**	**81.5%**

### Measurements, Statistics, and Applications

**Fig. 4:**
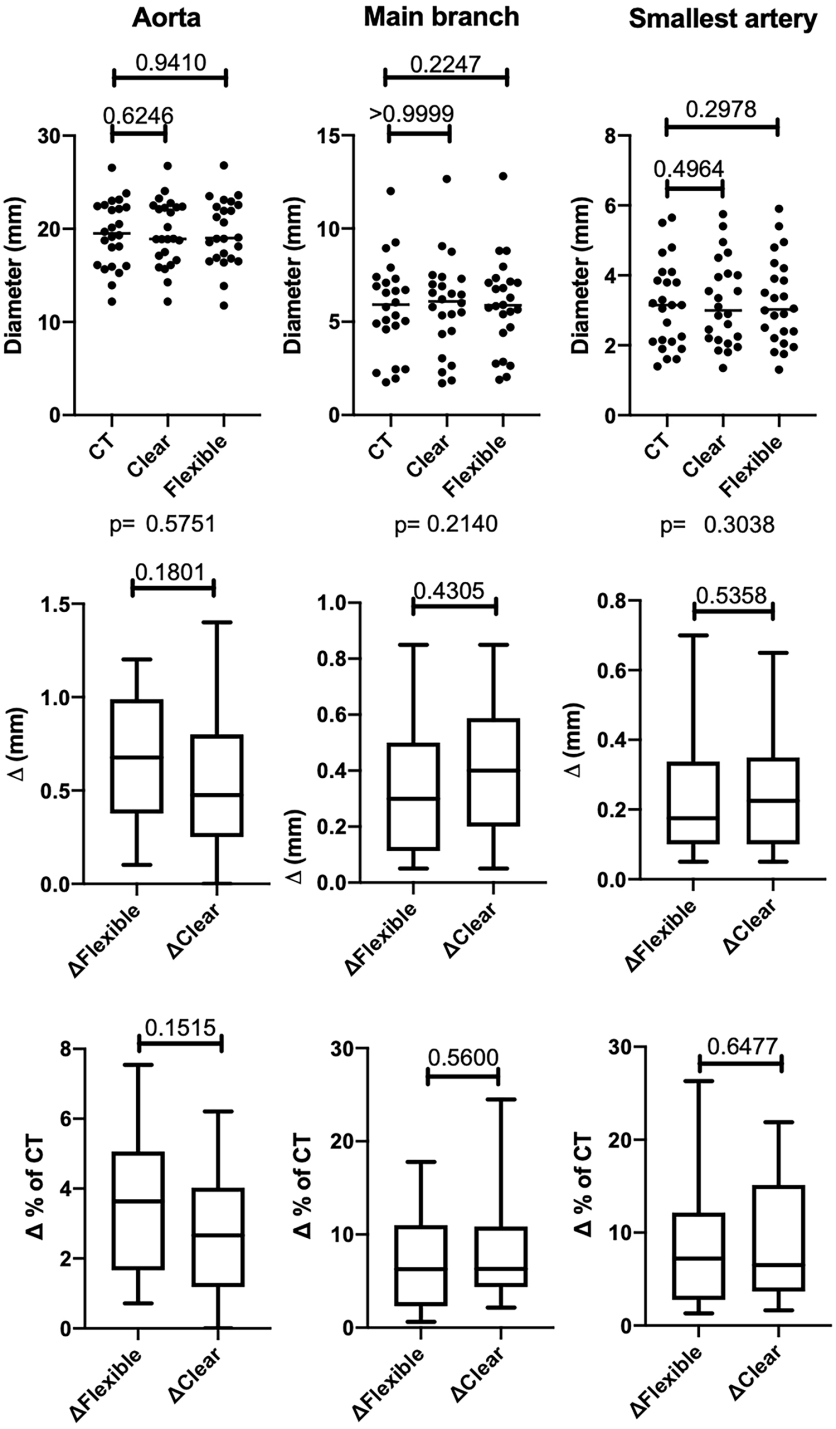
Box plots and scatter plots including the *p*-values for the mean diameters, the delta (Δ) in millimeter, and the delta in percent (%) measured on the CT scans of the patients, as well as the CT scans of the clear and flexible resin models in the subgroups aorta, procedure-relevant main branch, and smallest procedure-relevant artery

**Fig. 5:**
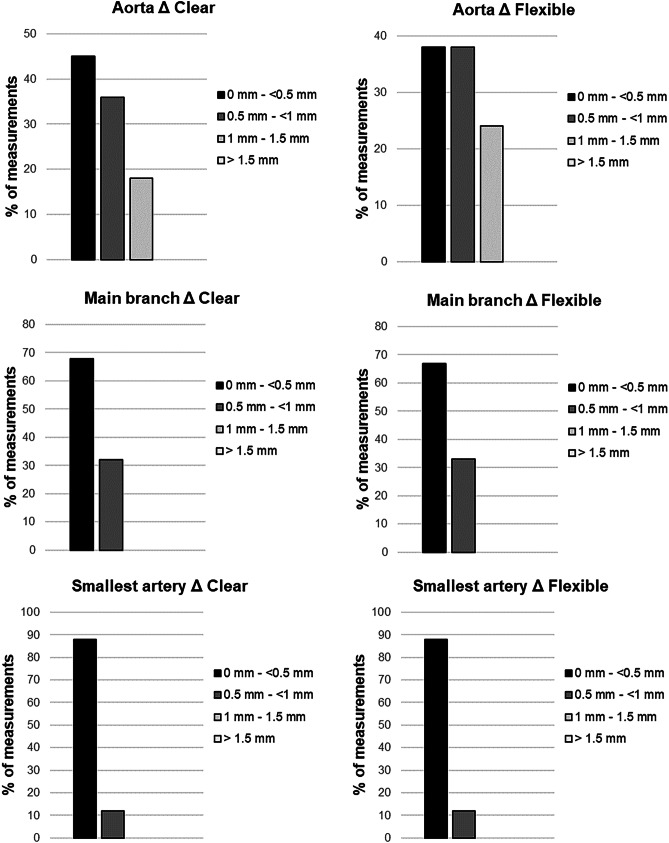
Distribution of deltas (Δ) in percent (%) with 0.5-mm increment for both materials and the subgroups aorta, main branch, and smallest artery. All deltas for the aorta were less than 1.5 mm and for main branch and smallest artery less than 1 mm. No statistical outliers were observed

**Fig. 6:**
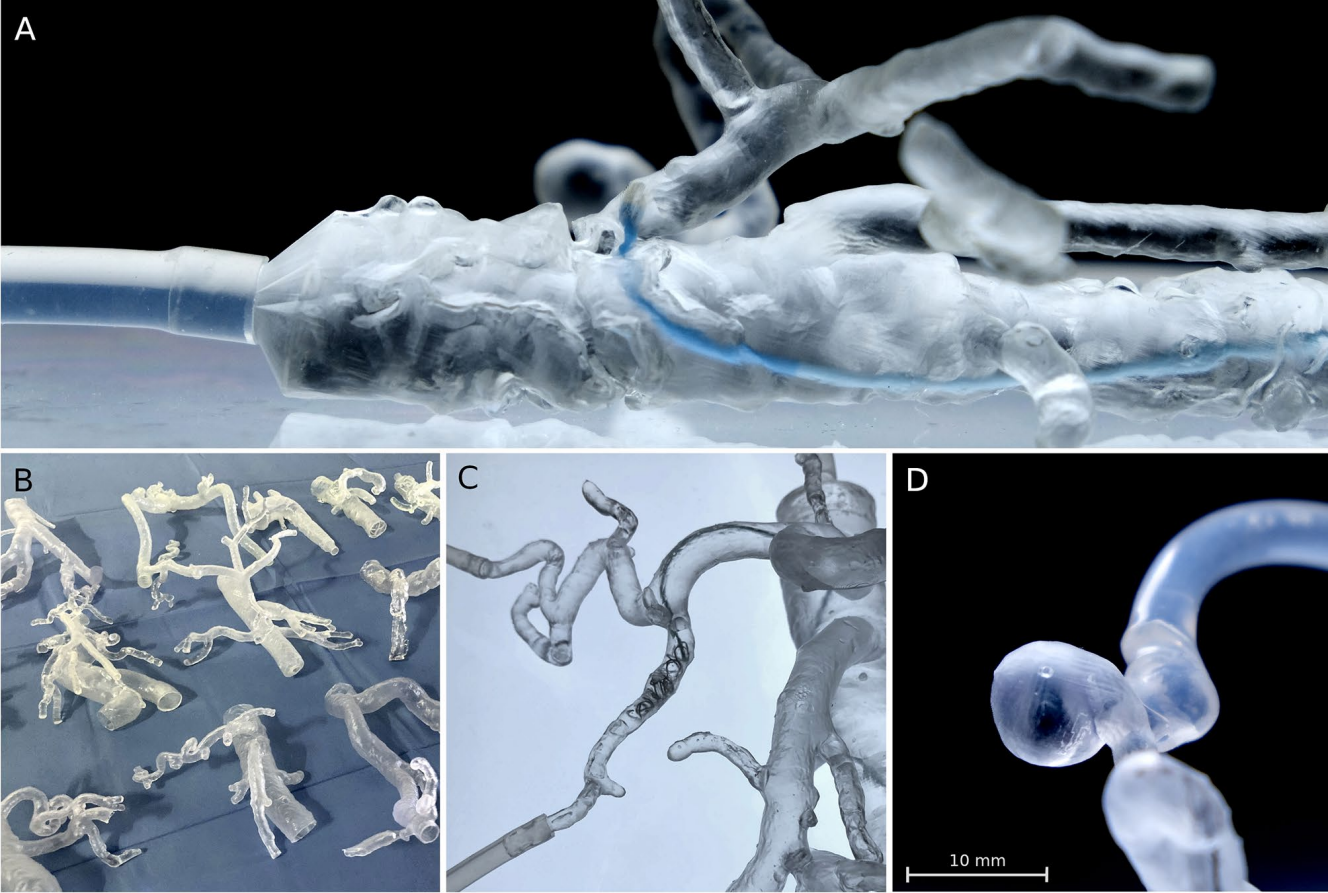
Applications of vascular 3D printing for IR. **A** The main catheter navigated to the celiac trunk in a severely calcified aorta (Note that calcifications were subtracted from the vessels; only the contrast enhanced vascular lumen was printed). **B** Examples of vascular models manufactured within this case series. **C** Simulation of the embolization of a gastroduodenal artery using a microcatheter system and coils. **D** Example of a 3D-printed splenic artery pseudoaneurysm caused by pancreatitis

Each successful case was measured at the three defined points for mean luminal diameters on its preprocedural CT and on the native CT scans of the clear and flexible resin models (216 measurements in total). The overall median absolute delta was 0.35 mm for clear resin and 0.32 mm for flexible resin; therefore, the printing accuracy for both materials was rated excellent (< 0.5 mm). No significant differences were observed in diameters using Friedman’s test to compare all diameter measurements of the original CT scan, the clear resin models and the flexible resin models in subgroups of aorta (*p* = 0.58), main branch (*p* = 0.21), and smallest artery (*p* = 0.3). In Dunn’s multiple comparison test the original CT scan was used as control group for the clear and flexible resin models in the subgroups aorta (*p* = 0.62 and 0.94), main branch (*p* = > 0.99 and 0.22), and smallest artery (*p* = 0.50 and 0.30). Also for the absolute (mm) and relative (%) deltas no significant differences were observed using the Wilcoxon matched-pair signed rank test in subgroups of aorta (*p* = 0.18 and 0.15), main branch (*p* = 0.43 and 0.65), and smallest artery (*p* = 0.54 and 0.56). The results are also demonstrated in Table 5 and visualized in Fig. 4. All deltas of the aorta were less than 1.5 mm and for the main branch and the smallest artery less than 1 mm; therefore, no absolute delta was rated as insufficient (Fig. 5). The largest aorta in this case series had a mean luminal diameter of 26.6 mm and the smallest artery 1.4 mm. As proof of concept, procedure simulations were performed with a peristaltic water pump and a smartphone camera for 2D projection using catheters, microcatheters, and guidewires, as well as embolic agents including coils and cyanoacrylate-based synthetic glue, demonstrated in Fig. 6. Video 1 illustrates the patient-specific procedure simulation of a superselective gastroduodenal artery embolization with coils in a pulsating flexible resin vascular model and visually summarizes the idea behind this study.

**Table 5:** Technical accuracy. Median luminal diameters, absolute deltas (Δ) in millimeter, and relative deltas in percent for aorta, main branch, and smallest procedure-relevant artery including the interquartile range (IQR). No significant differences were found between the printed models and their original CT (each *p* > 0.05) using Friedman’s test (1) and Dunn’s multiple comparison test for the diameters, as well as Wilcoxon matched-pair signed rank test for the deltas

	**Aorta**		**Main branch**		**Smallest artery**	
**Diameter**	**Median diameter**	***p***	**Median diameter**	***p***	**Median diameter**	***p***
Original CT	19.5 (± 6.3) mm	0.58 ^1^	5.9 (± 2.4) mm	0.21 ^1^	3.2 (± 1.8) mm	0.30 ^1^
Clear resin (V4)	18.9 (± 5.3) mm	0.62	6.1 (± 2.6) mm	> 0.99	3.0 (± 1.8) mm	0.50
Flexible resin (80A)	19.0 (± 5.5) mm	0.94	5.9 (± 2.5) mm	0.22	3.0 (± 1.6) mm	0.30
**Absolute delta Δ (mm)**	**Median delta (mm)**	***p***	**Median delta (mm)**	***p***	**Median delta (mm)**	***p***
CT vs. clear resin	0.48 (± 0.55) mm	0.18	0.40 (± 0.36) mm	0.43	0.23 (± 0.25) mm	0.54
CT vs. flexible resin	0.67 (± 0.54) mm		0.30 (± 0.36) mm		0.18 (± 0.21) mm	
**Relative delta Δ (%)**	**Median error (%)**	***p***	**Median error (%)**	***p***	**Median error (%)**	***p***
CT vs. clear resin	2.66 (± 2.70)%	0.15	6.31 (± 5.31)%	0.65	6.52 (± 10.28)%	0.56
CT vs. flexible resin	3.63 (± 3.17)%		6.27 (± 7.93)%		7.21 (± 8.46)%

Video 1. Embolization of a gastroduodenal artery to demonstrate the simulation setting with a silicone-like flexible resin model, connected to a peristaltic water pump. Note the pulsations of the vascular structures, caused by the flexible quality of the resin. To accomplish full embolization, coils might be combined with cyanoacrylate-based glue until stasis. To check for success, disappearing ink can be used as contrast agent. Li = Liver, Ki = Kidney, St = Stomach.

## Discussion

Interventional Radiology offers a wide range of potential educational and clinical applications for vascular 3D printing. Itagaki for example used a vascular model to treat a splenic aneurysm by testing and choosing catheter equipment preoperatively and using it as a reference intraoperatively. Before the actual procedure was performed, a guide catheter, base catheter, and microcatheter combination were selected and successfully used intraoperatively to reduce repeat angiograms for image orientation [18]. The 3D-printed visceral aneurysms have been described as technically accurate and aneurysm models were successfully used for simulation of endovascular treatments [19]. Simulation of EVAR has the potential to radically change endovascular surgery in the near future [20, 21], for example, by precisely locating the fenestration position preoperatively [22] or by facilitating EVAR-planning with complex neck anatomy [23]. Simulated 3D-printed CT-guided procedures were rated as realistic for training and learning purposes by interventional radiologists [24], and Javan and Zeman created an educational model for hepatobiliary interventions to demonstrate TIPS, percutaneous biliary drain, and cholecystostomy tube placement [25]. In the future, 3D printing of antibiotic and chemotherapeutic eluting catheters might lead to personalized IR [26].

This study is the first to evaluate the technical feasibility and accuracy of vascular SLA 3D printing with a novel transparent and biological tissue mimicking resin in IR [17]. 3D printing with this flexible resin was technically feasible in 81.5% in a consecutive case series of 27 embolizations. The printing accuracy of flexible resin was excellent with a median absolute delta (Δ) of 0.32 mm and a median relative delta of 4.78%, where excellent means absolute delta < 0.5 mm. In a total of 216 diameter measurements not a single absolute delta was rated as insufficiently accurate (Δ > 1.5 mm). No significant differences for diameters and deltas were observed in the subgroups aorta, main branch, and smallest procedure-relevant artery compared to a standard rigid clear resin with all *p*-values above 0.05 (Table 5), which means that flexible resin vascular models are technically as accurate as rigid standard clear resin models. However, standard clear resin had a slightly higher success rate with less print failures compared to the novel flexible resin (85.2% versus 81.5%), caused by one case which was not entirely printable with flexible resin: the pulmonary AVM due to partial rupture of the model while printing. This was caused by overhangs at an angle less than 10°, which made the overhang break off the model. The problem in this case did not occur with clear resin. Concerning that, the company mentions that there are slight variations about the minimum overhang angle at different resins. The same problem occurred in one other case but for both printing materials, probably due to extensive kinking of the arteries. Consecutively, some cases with extreme overhangs at small angles might be easier to print with clear resin at this stage (version 4), compared to the current version of flexible resin (version 1). Another technical limitation noticed in this case series were difficult segmentations caused by a lack of detail of the CT scan (e.g., no contrast agent in the procedure relevant artery), which certainly affects both materials likewise. To summarize, 4 out of 5 embolization cases over a half-year period were replicated technically accurate with complex vascular structures ranging from diameters of 26.6 mm (aorta) down to 1.4 mm (smallest arteries). Our findings correlate with Schicho et al. who stated SLA-printing as suitable and sufficiently reliable method for treatment planning in computer-assisted surgery [27]. Similarly, SLA 3D printing of vascular structures with biological tissue mimicking flexible resin is finally technically feasible with an end-user 3D printer and open-source software, as well as reliable concerning the technical printing accuracy. In a case-based approach, our results may improve the education and training of interventional radiologists when using such models for simulation of interventional procedures. A peristaltic fluid pump connected to the vascular models could be used to establish a closed circulatory system and the transparent material might enable radiation-free endovascular training of patient-specific procedures. Considering these aspects, this could also impact the procedural planning of interventions in the future, particularly in complex or non-routine cases.

A limitation is that the simulation setting was not evaluated yet, as this study is focused on the technical aspects of vascular 3D printing with flexible resin. In a next step, guidewire, catheter, and embolic agent behavior should be evaluated in a simulation setting, involving trainees as well as experienced interventional radiologists. As proof of concept, the supplementary video 1 demonstrates the usability of a patient-based flexible resin model used in a simulation setting with a peristaltic water pump. The main technical limitation of the manufacturing process is that the complexity of vascular models is limited by the resolution of the CT scan, compared to conventional angiography. Also a larger case series including MRI would have been of interest, but CT currently offers a higher resolution and, therefore, more details for vascular 3D printing.

Apparently, producing high-quality vascular models is not a one-click procedure, but rather a time-consuming, technical challenge. However, new technologies using deep learning with convolutional neuronal networks will improve model generation in the future by automatic segmentation of vasculature [28, 29] and maybe lead to even fully automatic generation of printable vascular models. Besides that, technical advances in additive manufacturing will also bring significant reduction of printing times, notably a technology initially described in the year 2015, called Continuous Liquid Interface Production (CLIP, Carbon). This innovative method uses digital light projection in combination with oxygen permeable optics which accelerates the manufacturing process of resin photopolymerization up to 100 times and allows fabrication of complex models within a few minutes, instead of hours [30]. The combination of artificial intelligence and newly arising 3D printing technologies is therefore particularly interesting for the field of radiology. Finally, automatized 3D printing for case-based simulations might be a chance for IR to progress towards a more personalized medicine.

## Conclusion

In conclusion, patient-specific vascular 3D printing with one of the first transparent biological tissue mimicking resins is technically feasible and accurate with an end-user 3D printer. In a consecutive case series of embolizations over a half-year period, 81.5% were printed successfully and with excellent technical accuracy. Future research has to evaluate the benefits of procedure simulations with such novel materials as 3D printing technologies are continuing to evolve. Vascular 3D printing in a case-base approach has the potential to impact education, training, and preprocedural planning in Interventional Radiology.

## Supplementary Information

Below is the link to the electronic supplementary material.

Supplementary file1 (MP4 89534 KB)

## Abbreviations

AVM: Arteriovenous malformation
CJP: Colorjet printing
CLIP: Continuous Liquid Interface Production
CT: Computed tomography
CTA: Computed tomography angiography
DLP: Digital light processing
EVAR: Endovascular aortic repair
FDM: Fused deposition modeling
HU: Houndsfield units
IR: Interventional radiology
MJP: Multijet printing
MPR: Multiplanar reconstruction
MRI: Magnetic resonance imaging
PJP: Polyjet printing
SIRT: Selective internal radiation therapy
SLA: Stereolithography 3D printing technology
SLS: Selective laser sintering
STL: Stereolithography file format
TIPS: Transjugular intrahepatic portosystemic shunt
